# Meningococcal purpura fulminans and severe myocarditis with clinical meningitis but no meningeal inflammation: a case report

**DOI:** 10.1186/s12879-019-3866-x

**Published:** 2019-03-12

**Authors:** Mehdi Hage-Sleiman, Nicolas Derre, Charlotte Verdet, Gilles Pialoux, Olivier Gaudin, Patricia Senet, Muriel Fartoukh, Mathieu Boissan, Marc Garnier

**Affiliations:** 1 Assistance Publique-Hôpitaux de Paris (APHP), Tenon University Hospital, Biochemistry Laboratory, 4 Rue de la Chine, 75020 Paris, France; 2 Assistance Publique-Hôpitaux de Paris (APHP), Tenon University Hospital, Medico-Surgical Intensive Care Unit, 4 Rue de la Chine, Paris, 75020 France; 3 Assistance Publique-Hôpitaux de Paris (APHP), Groupe Hospitalo-Universitaire Paris Est, Bacteriology Laboratory, 184 Rue du Faubourg Saint-Antoine, Paris, 75012 France; 4 Assistance Publique-Hôpitaux de Paris (APHP), Tenon University Hospital, Infectious Diseases Department, 4 Rue de la Chine, Paris, 75020 France; 5 Assistance Publique-Hôpitaux de Paris (APHP), Tenon University Hospital, Anatomopathology Laboratory, 4 Rue de la Chine, Paris, 75020 France; 6 Assistance Publique-Hôpitaux de Paris (APHP), Tenon University Hospital, Dermatology Department, 4 Rue de la Chine, Paris, 75020 France; 7 Sorbonne University School of Medicine, Paris VI, Paris, France; 8 INSERM UMR-S 938, Saint-Antoine Research Center, Sorbonne Université, Paris, France; 9 Assistance Publiuqe-Hôpitaux de Paris (APHP), Tenon University Hospital, Anaesthesiology and Intensive Care Medicine Department, 4 Rue de la Chine –, 75020 Paris, France

**Keywords:** Meningococcal disease, Purpura fulminans, Meningitis, *Neisseria meningitidis*, Myocarditis, Iloprost, Inflammation, Cerebrospinal fluid

## Abstract

**Background:**

During fulminant meningococcal septicaemia, meningococci are often observed in the cerebrospinal fluid (CSF) although the patients have frequently no meningeal symptoms. Meningococcal meningitis, by contrast, usually features clinical meningeal signs and biochemical markers of inflammation with elevated white blood cell count (pleiocytosis) in the CSF. Cases of typical symptomatic meningitis without these biochemical features are uncommon in adults.

**Case presentation:**

A 21-year-old male presented with meningococcal purpura fulminans and disseminated intravascular coagulation (DIC) associated with multiple organ dysfunction syndrome requiring hospitalization in the Intensive Care Unit. Despite typical meningeal clinical signs, lumbar puncture showed no pleiocytosis, normal glycorachia and normal proteinorachia, whereas the lactate concentration in the CSF was high (5.8 mmol/L). CSF culture showed a high inoculum of serogroup C meningococci. On day 2, after initial improvement, a recurrence of hypotension led to the diagnosis of acute meningococcal myocarditis, which evolved favourably within a week. During the hospitalization, distal ischemic and necrotic lesions were observed, predominantly on the fingertips, which were treated with local and systemic vasodilators.

**Conclusions:**

We report a rare case of adult meningococcal disease characterized by an intermediate form of meningitis between purulent meningitis and meningeal inoculation from fulminant meningococcal septicaemia, without classical signs of biological inflammation. It highlights the diagnostic value of CSF lactate, which may warrant administration of a meningeal dosing regimen of beta-lactam antibiotics. This case also demonstrates the potential severity of meningococcal myocarditis; we discuss its pathophysiology, which is distinct from other sepsis-related cardiomyopathies. Finally, the observed effects of vasodilators on the meningococcal skin ischemia in this case encourages future studies to assess their efficacy in DIC-associated necrosis.

## Background

Meningococcal disease encompasses several infectious syndromes. Despite the presence of *Neisseria meningitidis* in the cerebrospinal fluid (CSF) in both meningococcal meningitis and meningococcal septicaemia, these two diseases have distinct clinical presentations due to differences in their pathophysiology; notably, the compartmentalization of the bacterial injury and the inflammatory response [1–3]. Meningococcal meningitis is similar to other forms of acute purulent meningitis, with high *N. meningitis* inoculum in the CSF causing meningeal inflammation and typical clinical signs of meningitis [1–3]. By contrast, in fulminant meningococcal septicaemia, *N. meningitis* multiplies very quickly in the blood, causing extensive endotoxemia and, in the most severe forms, shock and multiple organ dysfunction syndrome [2, 3]. In this latter form, meningococcemia may be responsible for haematogenous inoculation of the CSF, which leads, in the great majority of cases, to a low level *N. meningitis* meningeal inoculum, which neither triggers significant meningeal pleiocytosis nor causes clinical signs of meningitis [2]. Meningococcemia, classically, may also be responsible for pericardial infection, arthritis, pneumonia, conjunctivitis, panophtalmitis, and infections of the genito-urinary tract [1, 2].

We report here a case of meningococcemia with purpura fulminans, septic shock and clinically symptomatic meningitis, yet with no sign of CSF inflammation, associated with acute severe myocarditis.

### Case presentation

**Fig. 1:**
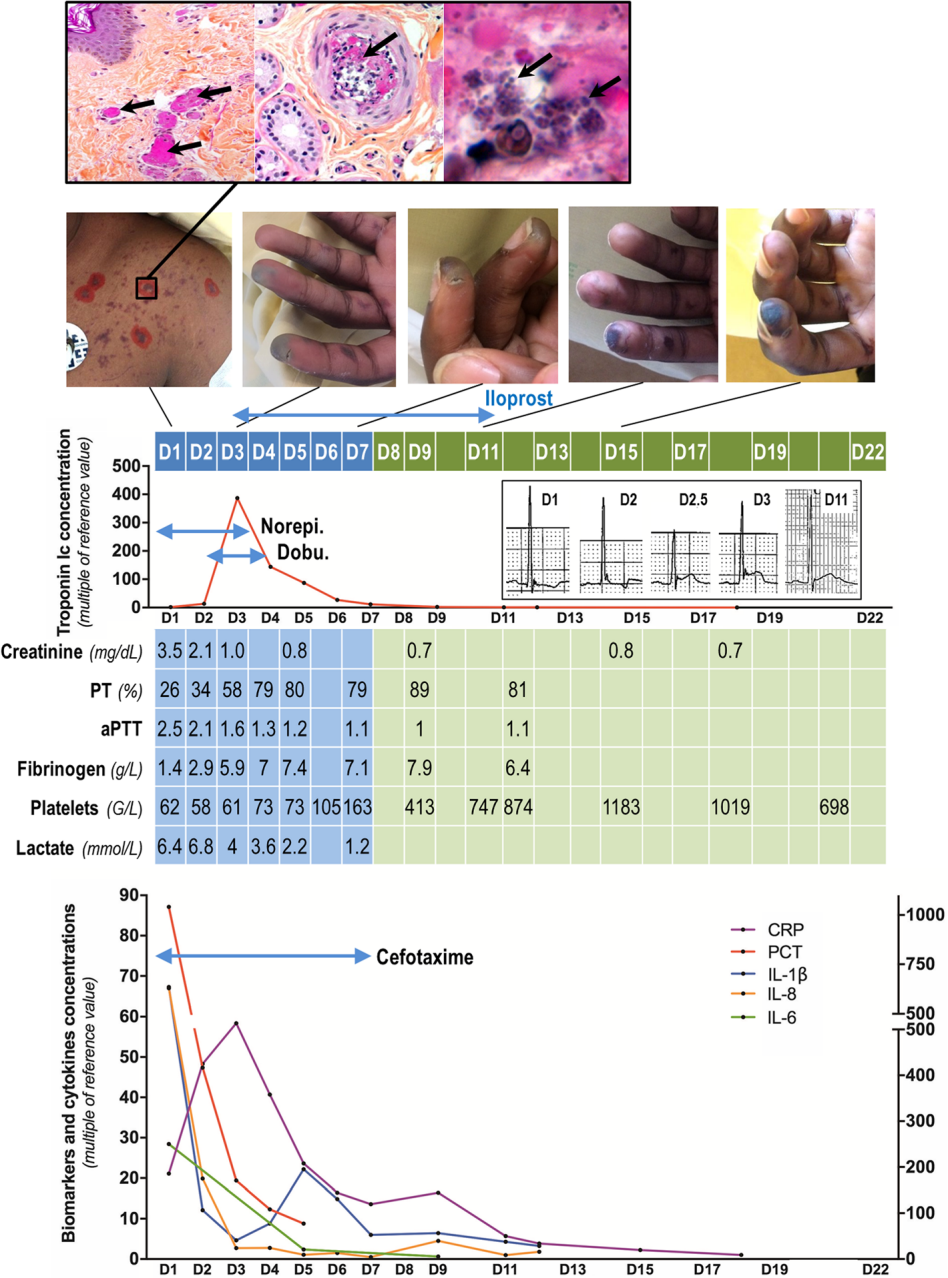
Summary of the patient’s clinico-biological course. At admission (D1), the patient presented diffuse purpuric lesions (photo, left) that, upon pathological analysis (upper panels), showed thrombosis of nearly all dermal capillaries (left; fibrin is stained pink with hematoxylin–phloxine–saffron stain [arrows]; × 100) as well as several deep dermal arterioles (middle [arrow]; × 200), and the presence of cocci inside the thrombi (right [arrows]; × 800). Analyses of DIC (prothrombin time [PT]), activated partial thromboplastin time (aPTT), fibrinogen and platelets are shown in the table (blue columns indicate care in the ICU; green columns indicate care in the infectious diseases department). The patient presented distal digital ischemia, which was treated from D3 to D11 with Iloprost with a favourable local outcome; final necrosis being limited to the second fingertip (sequential photos of fingers). Upon admission, septic shock was treated with norepinephrine infusion (Norepi*.*). On D2, the patient presented an acute myocarditis with a hypersensitive troponin Ic peak (red graph) of around 390-fold the reference value and an elevation of the ST segment in the infero-lateral area beginning at D2.5 (captures of the V5 lead on the ECG), requiring dobutamine infusion (Dobu*.*) for 3 days, with a favourable outcome; troponin Ic concentration returned to normal on D9. The septic shock was complicated by multiple organ failure (see creatinine and lactate values in the table) and systemic biological inflammatory syndrome (lower graph) with an early dramatic increase of blood procalcitonin > 1000-fold the reference value (PCT, red curve, right axis) and a delayed 60-fold increase of C-reactive protein (CRP, purple curve, left axis). Concomitantly, blood concentrations of interleukin-1β (IL-1β, blue curve, left axis), IL-8 (orange curve, left axis) and IL-6 (green curve, right axis) were hugely increased at D1. They returned to normal upon treatment with cefotaxime from D1 to D7, as indicated

A 21-year-old male, who previously suffered only from intermittent asthma, was admitted to the emergency room in March 2018 with a one-day history of headache, nausea, sore throat, and generalised muscle ache. An initial consultation with the family physician had diagnosed influenza but shivers, photophobia, and testicular pain appeared 24 h later. Subsequent physical examination found new purpuric lesions on the trunk and upper limbs (Fig. 1) leading to admission to hospital.

At admission, the patient’s blood pressure was 121/47 mmHg, heart rate was 116 bpm, oxygen saturation was 94% in room air, and his temperature was 38.4°C. He was slightly drowsy with a Coma Glasgow Scale score of 13, with left parietal headache, nausea and neck stiffness. Cardiovascular, pulmonary and abdominal examination was normal. Laboratory analysis of blood samples revealed high levels of C-reactive protein (106 mg/L), hyperleukocytosis (24 × 10^9^ white blood cells/L, of which 94% were neutrophils), and acute non-obstructive renal failure (3.46 mg/dL serum creatinine, corresponding to creatinine clearance of 24 mL/min).

Blood cultures were taken and a lumbar puncture was performed, followed immediately by intravenous (IV) administration of 2 g cefotaxime. The CSF was crystal clear and no hyper-pressure was observed upon puncture of the dura mater. Biochemical analysis of the CSF revealed normal glycorrachia (3.0 mmol/L, with 4.8 mmol/L glycaemia), normal protein content (0.22 g/L) and elevated levels of lactate (5.8 mmol/L). Cyto-microbiological analysis found no CSF pleiocytosis (6 leukocytes/mm^3^) and the absence of bacteria as determined by Gram staining.

The patient was admitted to the intensive care unit (ICU) with a diagnosis of purpura fulminans with uncertain meningitis. During the following 12 h, multiple organ dysfunction syndrome progressively appeared with the following features: disseminated intravascular coagulation (DIC) [elevated prothrombin time (PT) (26%), elevated activated partial thromboplastin time (aPTT) (2.54), low fibrinogen (1 .4g/L), thrombopenia (62 × 10^9^ platelets/L), elevated D-dimers (> 10.000 ng/mL) and low factor V (21%)]; severe hypotension resistant to 20 mL/kg fluid resuscitation and requiring treatment with 0.4 μg/kg/min norepinephrine; non-obstructive acute renal failure; acute lung injury with mild pulmonary oedema upon chest X-ray and no cardiac failure upon the first echocardiographic examination (left-ventricular ejection fraction (LVEF) 70%), requiring oxygen delivery through a mask up to 9 L/min flow, and metabolic acidosis (pH 7.28, lactate 6.4 mmol/L). In addition, plasma procalcitonin (PCT) levels were very high (521 μg/L). Encephalic computerised tomography (CT) scan and magnetic resonance imaging (MRI) ruled out the presence of a pharyngeal or cerebral abscess, cerebral thrombophlebitis, sinusitis, mastoiditis, and ethmoiditis. The patient was treated IV with 250 mg/kg/day cefotaxime.

*Neisseria meningitidis* was first identified in the blood cultures after 15 h, confirming the diagnosis of meningococcemia with purpura fulminans and shock. Numerous *N. meningitidis* colony-forming units were then identified in the CSF cultures 24 h after sampling. Furthermore, pathological examination of skin biopsies taken from purpuric areas revealed thrombosis of all the dermal capillaries associated with the presence of cocci in several vessels (Fig. 1). The *N. meningitidis* strain isolated belonged to serogroup C and was fully susceptible to penicillin (minimum inhibitory concentrations for penicillin, amoxicillin and ceftriaxone of 0.047, 0.125, and < 0.016 mg/L, respectively). The patient had never been vaccinated against meningococcus. Human immunodeficiency virus serology was negative.

Organ failure improved by the second day after admission. Oxygen delivery was decreased to 4 L/min and the norepinephrine infusion rate reduced to 0.2 μg/kg/min. Creatinine serum levels decreased to 2.1 mg/dL (estimated clearance of 40 mL/min), haemostasis parameters improved (PT 42%, aPTT 1.85, fibrinogen 4.5 g/L), and the blood lactate concentration decreased to 5.7 mmol/L (Fig. 1). A recurrence of hypotension, however, led to the diagnosis of acute myocarditis upon echocardiography, with decreased LVEF (40%), diffuse left-ventricular hypokinesia, and low left-ventricular output (2.4 L/min/m^2^ with aortic velocity–time integral of 13.5 cm). An electrocardiogram revealed an elevation of the ST segment in the infero-lateral area (Fig. 1). Blood levels of hypersensitive troponin Ic increased rapidly to reach a peak of > 13,000 ng/L 44 h after admission, and then decreased and normalized within 14 days. Hypotension was corrected by IV infusion of 10 μg/kg/min dobutamine for 36 h until left-ventricular function was completely restored. A myocardial MRI scan performed at day 8 showed no residual segmental or global left-ventricular dysfunction, no perfusion defect, and no abnormal contrast enhancement after injection of gadolinium.

All organ dysfunctions resolved during further treatment in the ICU. Nevertheless, distal hypoperfusion remained, in particular, in the distal phalanges of both hands. To avoid necrosis of the fingers, the patient was treated for 9 days with systemic (Iloprost 2 ng/kg/min by continuous IV) and local (Trinitrine patches 10 mg/day applied to all distal phalanges) vasodilators. Dermal necrosis occurred only on the pulp of the left index finger, bilaterally on the pulp of the big toes, the left ear lobe and the foreskin. Cefotaxime was discontinued after 7 days. The patient was transferred to the infectious disease ward 7 days after admission into the ICU. He was circumcised at day 12 to treat the foreskin necrosis and was finally discharged from the hospital at day 22 after favourable evolution of all necrotic lesions.

## Discussion and conclusions

This patient suffered from purpura fulminans complicated by DIC and multiple organ dysfunction syndrome, including severe acute myocarditis. Three aspects of this case make it instructive.

First, although typical clinical signs of meningitis were observed and numerous *N. meningitidis* bacteria were isolated from CSF cultures, the biochemical and cytological analyses of the CSF did not identify meningeal inflammation. This discrepancy has been described previously in 0.5–12% of cases of paediatric meningitis [4–6]. It is rare, however, in non-neutropenic and non-immunocompromised adults [7–9]; in most reported cases, adults suffering from meningitis with normal initial CSF analysis had an incomplete clinical presentation with, in particular, a lack of neck stiffness [5, 10, 11]. Furthermore, in most reported cases where pleiocytosis was absent, CSF analysis showed moderate hyperproteinorachia or hypoglycorachia [8], neither of which was seen in our case. In several cases where no bacteria were found in cultures of the CSF sampled by a first lumbar puncture, subsequent lumbar puncture later found high pleiocytosis and the presence of bacteria [8], suggesting that the first CSF sample was taken too early to observe the meningeal inflammatory response. This early sampling might explain the lack of bio-cytological indicators of inflammation in the CSF of our patient; however, culture of the CSF sample taken at the first lumbar puncture found a high inoculum of *N. meningitis* bacteria. Thus, our case appears to be an intermediate form of meningococcal disease between purulent meningitis (indicated by the typical clinical signs and CSF microbiological cultures) and paucisymptomatic meningeal inoculation from fulminant meningococcal septicaemia (indicated by the cyto-biochemical CSF analysis). Despite the lack of cyto-biochemical signs of meningeal inflammation, the elevated level of CSF lactate seen in this case was a warning sign of bacterial meningitis. A previous prospective cohort of patients with meningitis and negative CSF upon Gram staining reported that CSF lactate concentration was the best marker to discriminate bacterial from aseptic meningitis [12]. A CSF lactate concentration > 3.8 mmol/L was 94% sensitive, 97% specific, and had 82% positive and 99% negative predictive values to indicate the bacterial origin of the meningitis [12]. This finding was confirmed by two meta-analyses that found that CSF lactate concentration was the best diagnostic tool to discriminate bacterial from aseptic or viral meningitis, particularly when lumbar puncture was performed prior to antibiotic administration [13, 14]. Thus, despite the absence of pleiocytosis, hypoglycorachia, or hyperproteinorachia, our patient’s CSF lactate concentration of 5.8 mmol/L led us to maintain empirical cefotaxime administration at 250 mg/kg/day. When considered together with the previous reports mentioned above, the current case should encourage physicians not to discard a diagnosis of acute bacterial meningitis on the basis of a normal CSF cell count, and/or Gram staining but to be attentive to CSF lactate values in cases of suspected bacterial meningitis, as recommended recently [9, 15].

Second, this patient presented a delayed acute myocardial dysfunction, which occurred 36 h after admission to hospital. An acute coronary syndrome due to the occlusion of a coronary artery was ruled out based on clinical and echocardiographic data and was definitively disproved by cardiac MRI. This acute myocardial dysfunction might then be explained by at least three other pathophysiological causes: septic shock, meningococcal-related myocarditis and DIC-related myocardial injury. Sepsis-related myocardial dysfunction occurs in about one third of septic patients and, in 30% of cases, it occurs after the first 24 h of septic shock management, consistent with this case [16]. The pathophysiology of septic cardiomyopathy is complex and still a subject of debate, but a direct effect of inflammatory cytokines, myocardial inflammatory oedema, increased oxidative stress, and decreased expression of adrenergic receptors likely contribute to the malfunction of the myocardium [17]. Notably, IL-1β, which was dramatically elevated in this case (Fig. 1), is reported to cause a concentration-dependent depression of myocardial cell contractility [18]. Meningococcal-related acute myocarditis is probably an underdiagnosed complication of meningococcal disease. Indeed, myocarditis has been reported in 30% of adult patients suffering from the most severe forms of invasive meningococcal disease requiring ICU admission [19]. In these cases, left-ventricular dysfunction may be due to direct bacterial injury of the myocardium and to the meningococci-induced inflammatory response [20, 21], which may be cytotoxic for the myocardium [22]. In addition, meningococcal disease is specifically associated with high production of IL-6, as we observed in this case (Fig. 1), causing cytokine-mediated myocardial depression [23, 24]. Thus, systemic meningococcal spread and the associated IL-6 overexpression may have contributed to the myocardial dysfunction observed in our patient. Finally, it has been reported that the extent of myocardial dysfunction and levels of troponin correlate with the severity of the meningococcal coagulopathy in paediatric ICU patients [25]. As this patient presented severe DIC, it is also possible that distal myocardial microthrombi contributed to the cardiac dysfunction.

The third informative aspect of this case is the use of vasodilators to reduce the extent of skin necrosis. Distal ischemia and necrosis are common complications of the purpura fulminans coagulopathy [3, 26] and severe vasoconstriction and intravascular thrombosis have previously been associated with poor prognosis in meningococcal shock [27]. Several pathophysiological factors contribute to peripheral vasoconstriction, capillary thrombosis and skin necrosis during meningococcal disease: deficiency in the production of prostacyclin (a potent inhibitor of platelet aggregation and powerful vasodilator) by the vascular endothelium [28], enhanced endothelial activation and production of leucocyte adhesion molecules [29], and increased systemic plasminogen activator inhibitor-1 (PAI-1) levels contributing to reduce fibrinolysis and promote DIC [30]. Iloprost is an analogue of prostacyclin whose pharmacological effects include vasodilation of arterioles and venules, inhibition of platelet activation, and induction of fibrinolysis. Thus, its use may contribute to the treatment of several pathophysiological processes underlying skin necrosis. No prospective studies have assessed its efficacy to reduce the extent of meningococcal-related skin necrosis, however. Only two old paediatric case reports of prostacyclin use to this end are available [31, 32]. In the current case, we administered Iloprost according to the protocol used for Raynaud’s Syndrome and in association with local treatment with trinitrine patches. Tolerance was good and there was no need to reintroduce catecholamine. Local evolution was favourable without recourse to surgery except to treat a hyperalgic ulcerative necrotic lesion of the foreskin. Although the efficacy of Iloprost for the treatment of skin necrosis cannot be assessed from this one case, our observations are encouraging for future studies that would evaluate the potential efficacy of systemic and/or local vasodilator treatments in preventing and/or improving the evolution of skin necrosis.

## Abbreviations

aPTT: Activated partial thromboplastin time
CSF: Cerebrospinal fluid
CT: Computerised tomography
DIC: Disseminated Intravascular Coagulation
ICU: Intensive care unit
IL: Interleukin
IV: Intravenous
LV: Left ventricular
MRI: Magnetic resonance imaging
PCT: Procalcitonin
PT: Prothrombin time
